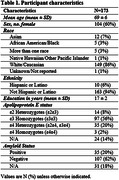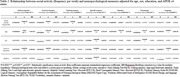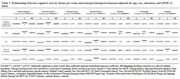# Association of lifestyle activities with cognitive function and Alzheimer’s disease CSF biomarkers: an examination of cognitively normal adults in the Stanford Aging and Memory Study (SAMS)

**DOI:** 10.1002/alz.084160

**Published:** 2025-01-09

**Authors:** Olivia Lu, Elizabeth Mormino, Zihuai He, Valerie A Carr, Alexandra N. Trelle, Christina B. Young, America Romero, Hillary Vossler, Jennifer Park, Irina Anna Skylar‐Scott

**Affiliations:** ^1^ Stanford University, Palo Alto, CA USA; ^2^ Stanford University, Stanford, CA USA; ^3^ San Jose State University, San Jose, CA USA; ^4^ Stanford University School of Medicine, Stanford, CA USA

## Abstract

**Background:**

It is increasingly clear that delaying the onset of Alzheimer’s disease (AD) dementia by several years can meaningfully lower its prevalence. The goal of the present study is to examine the relationship between lifestyle activities and cognition function as well as cerebrospinal fluid (CSF) biomarkers of AD to determine whether these activities can serve as protective factors for AD resistance and resilience.

**Methods:**

173 cognitively normal older individuals (mean ± SD, 69 ± 6.4 years) were recruited to the Stanford Aging and Memory Study (SAMS) and completed the Community Healthy Activities Model Program for Seniors (CHAMPS) questionnaire regarding current social, cognitive, and physical activity (Table 1). They also underwent APOE genetic testing and a detailed neuropsychological evaluation. The following cognitive domains were evaluated after conversion to z‐scores: global cognition (cognitive composite), executive function, working memory, attention, episodic memory, visuospatial function, and language (see Table 2 for definitions). 127 participants completed lumbar punctures, and levels of Aβ‐40, Aβ‐42, p‐tau181, and total tau were measured in the CSF. Cross‐sectional regression models included age, sex, years of education, and APOE status as co‐variates. Benjamini‐Hochberg corrections for multiple hypotheses were completed.

**Results:**

There was a significant association between social activity (frequency/week) and global cognition (β = 0.20, p = 0.03), executive function (β = 0.15, p<0.05), and working memory (β = 0.26, p = 0.01) but not episodic memory, visuospatial function, or language function. There was also a significant association with attention prior to correction for multiple hypotheses (β = 0.17, p = 0.04) but not afterward (Table 2). Additionally, there was a significant association between cognitive activity (hours/week) and global cognition (β = 0.19, p = 0.03) as well as executive function (β = 0.25, p = 0.007) but not with other cognitive domains tested (Table 3). There was no association between light or moderate caloric expenditure and cognitive measures. There was also no significant relationship between CSF biomarkers and levels of social, cognitive, and physical activity.

**Conclusions:**

In a well‐characterized cohort of cognitively normal older adults, higher levels of social and cognitive activity were associated with higher cognitive scores on tasks of executive function but not episodic memory. The mechanism mediating this relationship appears to be independent of both Aβ and tau burden.